# Epicarin in the Treatment of Scabies

**Published:** 1901-02

**Authors:** W. E. A. Wyman

**Affiliations:** Milwaukee, Wis.


### EPICARIN IN THE TREATMENT OF SCABIES.

By W. E. A. Wyman, M.D.V., V.S.,
MILWAUKEE, WIS.

In the treatment of mange many agents are employed. Of these picis liquida, creolin, creosote, lysol, Peruvian balm, sulphur, iodine, and salicylic acid are more frequently used, either alone or in combination with each other. More recently the Farbenfabriken of Elberfeld Company, New York City, have produced an exceedingly effective, penetrating, non-irritating, non-poisonous, inodorous, and last, but not least, a neat, antiscabious drug termed epicarin—a naphtol preparation.

The presence of more or less eczema following constant scratching in cases of mange often renders its treatment in fine-skinned dogs rather difficult with any of the first-named agents. As a rule, only a part of the skin of the body can be treated at a time, as the possibility of an absorption followed by toxic states always presents itself. While not impossible, it is at least expensive and often difficult to bandage the patient to prevent his licking the application. Another objection, and a serious one, is the persistent and disagreeable smell connected with most compounds, and their oleaginous nature, spoiling carpets, furniture, etc.

The writer has now treated sixty-three individual cases of mange with epicarin, and considers himself entitled to express positive criticism on this product of the Elberfeld Company. Fifty-one were in the dog (sarcoptes squamiferus); four dogs with acarus folliculorum; five cats with sarcoptes minor; two horses with sarcoptes equi, and one cow with dermatocoptes communis.

In order to give epicarin no undue credit, microscopical examinations were made in all of these sixty-three cases. The dog with supposed sarcoptes mange is placed in a warm room, and as soon as the heat has the desired effect—that is, the animal begins to scratch persistently—a bit of the diseased skin is scraped until the blood oozes; large scales are cleared up with a potash solution, 1 : 5, or placed upon a warm piece of black paper and then examined. Dermatocoptes communis of the ox is easily detected, as it penetrates only the epidermis. The pustulous form of acarus folliculorum presents a clear clinical picture, but may occasionally be confounded with acne or furunculosis. Even in the squamous type the parasites can always be squeezed out by gliding with the back of the knife, with fair pressure, on the diseased parts, thus differentiating it readily from eczema.

The epicarin treatment is executed as follows: The mangy dog is not clipped unless the amount of infected skin demands it. Liquid German soap is liberally applied to the whole of the body and the animal placed in a bath containing ½ per cent. of creolin. When dry the following formula, suggested by Frick, is thoroughly rubbed in all over the body with a soft brush:

| | |
|---|---|
| ℞.—Epicarin . . . . . . . . | 10 |
| Ol. ricini . . . . . . . . | 10 |
| Spiritus . . . . . . . . | 100.—M. |

The soap-creolin bath, followed by an epicarin application, is repeated every fifth day. Three applications invariably prove sufficient to cure the case.

Cats, unless absolutely necessary, are not bathed, the deciding factor being the extent of the diseased skin area, and are, therefore, simply brushed with the epicarin liniment every three or four days.

Horses and cattle are first scrubbed with Parke, Davis & Co.'s germicidal soap, followed, when dry, with the epicarin liniment, the process to be repeated every three or five days, depending on the severity of the case.

The treatment of acarus folliculorum mange, as is well known, is only too often accompanied with negative results. The four cases treated with epicarin made a complete recovery. First, the dog is clipped; next, each pustule is opened and its contents removed. Now, the animal is given a 1 per cent. creolin bath for two or three minutes. When dry the epicarin liniment is thoroughly applied. This creolin bath, followed by epicarin, is repeated every third day. Of course, a strongly supportive dietetic regimen is a necessity.

Epicarin thus used affected the patients as follows: The dogs appeared uneasy for three to five minutes; after that little or no itching was observed. All patients were given three applications. All recovered, and none were returned subsequently.

Those cases of acarus folliculorum—two with decided sclerosis of the skin—were under hospital treatment from eighteen days to eight weeks. All recovered.

The five cats did not seem to mind the epicarin application at all. They received three applications, and recovered.

The cow got only one brushing with epicarin, as the owner broke the bottle. She also recovered completely.

One horse apparently recovered after three applications, but was returned itching one month after the last treatment had been given. It received three more brushings with epicarin, and has remained well since. The second horse—a case of generalized mange—was experimentally treated in the above-described manner for two months, when it was killed, being distinctly cachectic. But even in this case epicarin proved itself a powerful antiscabious agent.

SUMMARY. Epicarin removes itching almost immediately, and is well borne by all classes of patients. It is non-poisonous, non-irritating, inodorous, not as sticky or greasy as most of the agents formerly employed in the treatment of mange, penetrating, and thus effective. Its cost is reasonable.

Properly employed, the writer considers it a permanent and exceedingly valuable antiscabious remedy in canine and feline practice, quite indispensable in the management of cases to be treated at the home of the owner.

